# Cu-Ag Nanocomposite Pastes for Low Temperature Bonding and Flexible Interlayer-Interconnections

**DOI:** 10.3390/nano12234241

**Published:** 2022-11-29

**Authors:** Yin-Chi Lu, Wei-Hsun Liao, Ting-Jui Wu, Kiyokazu Yasuda, Jenn-Ming Song

**Affiliations:** 1 Department of Materials Science and Engineering, National Chung Hsing University, Taichung 402, Taiwan; 2 Division of Materials and Manufacturing Science, Graduate School of Engineering, Osaka University, Osaka 565-0871, Japan; 3 Innovation and Development Center of Sustainable Agriculture, National Chung Hsing University, Taichung 402, Taiwan

**Keywords:** nanocomposite, low temperature bonding, flexible substrate, reliability

## Abstract

Cu-Ag composite pastes consisting of carboxylate-capped Ag nanoparticles, spray-pyrolyzed Ag submicron particles, and copper formate were developed in this study for low-temperature low-pressure bonding. The joints between the Cu, Ni/Au, and Ag finished substrates can be well formed at temperatures as low as 160 °C under a load pressure of 1.6 MPa. The joints with Cu substrates possess 18.0 MPa bonding strength, while those with Ag surface finish could be enhanced to 23.3 MPa. When subject to sintering under 10 MPa at 160 °C, the electrical resistivity of the sintered structure on metal-coated polymeric substrates was around 11~17 μΩ-cm and did not differ too much when subjected to harsh reliability tests such as mechanical bending and thermal cycling tests, as well as electrical current stressing. This low-temperature, low-pressure nanocomposite paste shows great potential as interconnect materials for microelectronics or flexible device assembly.

## 1. Introduction

In response to anxiety over human health and the environment, most countries have established legislation to ban the use Pb from electronic products [1]. Researchers have searched for alternatives to Pb-containing solders for two decades [2]. Pb-free eutectic solders, such as Sn-In, Sn-Bi, Sn-Ag, and Sn-Cu, have their limitations [3]. For instance, their die attachment for chips cannot withstand high operating temperatures. Electrically conductive adhesives (ECAs) bonding technologies are also regarded as a Pb-free alternative and have been actively applied for microelectronic packaging applications [4]. All ECAs consist of a polymer matrix with submicron-sized electrically conductive fillers [5]. Despite the low bonding temperature (<200 °C), they provide insufficient mechanical strength and thermal and electrical conductance. This is because the sticky components just hold the ECAs together and the conductive particles are only in contact with each other, instead of having robust links.

The size-dependent properties of nanomaterials, such as their highly specific surface area and decreased melting point, have been maximized for their practical use. Au, Ag, and Cu nanoparticles have been applied in the fabrication of interconnectors for microelectronics, including flat panel displays and flexible and wearable devices [6,7,8,9,10]. Nanoparticles can be sintered at low processing temperatures, and thus, the interconnections formed can withstand high operating temperatures. Ag has been well-studied and widely adopted due to its good electrical conductivity. However, it still exhibits the drawbacks of high cost and poor electrochemical migration resistance. Cu nanoparticles have recently been proposed as a replacement. Cu NPs, however, have a huge problem—easy oxidation.

To solve this problem, Cu-Ag alloy, Cu@Ag core shells, as well as a mixture of Cu and Ag particles have been developed [11,12,13,14,15]. The combination of Cu and Ag exhibits many advantages, such as improved electrochemical migration resistance compared with Ag [11,12] and superior anti-oxidation properties than Cu. Improvement in thermal stability has also been demonstrated [13,14,15].

In this study, a composite paste designed for a low-load and low temperature bonding process comprised of nano- and submicron-sized Ag particles with copper formate is proposed. The purpose is to mix metallic particles of various sizes together to fill the interstices between bigger particles with smaller ones [16]. Adding copper formate can further fill the gaps and form a more solid structure with improved electrical conductance [17]. A recent report verified that copper formate additives in copper nanoparticle pastes decomposed into new active nano copper at 300 °C and were able to strengthen the Cu-Cu joints bonded in the process [18]. The goal of this study is to robustly bond the samples at a temperature of 160 °C, which vulnerable polymeric substrates can withstand. In view of their future use as interconnections with flexible substrates and components, the joints between two Cu cladded PI substrates (PI: polyimide) were also prepared using the developed composite pastes. Reliability was tested using mechanical and thermal cycling tests (TCT). Current stress was also evaluated. To clarify the surface finish effects, Ni/Au and Ag coating onto Cu foils were also adopted.

## 2. Experimental Procedures

### 2.1. Fabrication of Nanocomposite Pastes

Composite pastes for a low-load and low temperature bonding process (designated as LNCP) consisting of nano- and submicron-sized Ag particles and copper formate were prepared. Ag nanoparticles were protected by self-assembled carboxylate monolayers with six carbon atoms (hexanoic acid). The synthetic method has been introduced elsewhere [19], but the detailed procedure is as follows. First, 1.4156 g (8.33 mmol) AgNO_3_ was added to a toluene solution (31.69 mL) containing hexanoic acid (1.861 g, 16.67 mmol) and 0.5656 g PVP (poly-vinylpyrrolidone, as the dispersant), and the mixture was stirred vigorously. Then, 1.65 mL (16.67 mmol) n-butylamine was added dropwise over a period of 2.5 min to the solution. After the mixture had been stirred for a further 3.5 min, the solution became milk white. A 50 mL aqueous NaBH_4_ solution (0.158 g, 4.17 mmol) as a reducing reagent was added dropwise over 15 min with vigorous stirring. After the addition of the reductant, the solution was stirred for 1 h. Products were precipitated by the addition of 200 mL acetone. Following centrifugation, resuspending in methanol, precipitating and washing with acetone, the desired dark-brown silver nanoparticles were obtained.

Thermal spray pyrolysis was used to prepare submicron-sized Ag particles. The synthesis procedure was as follows. Silver acetate precursor (99%, AgC_2_H_3_O_2_, Alfa Aesar, Ward hill, MA, USA) was used to prepare silver particles using a laboratory-scale spray pyrolysis electrostatic deposition system. In the spray pyrolysis process, the precursor aqueous solution (0.06 M) was atomized into fine droplets using an ultrasonic nebulizer at 1.65 MHz. An air flow with a controlled flow rate carried the droplets into the heated tubular reactor with three zones: pre-heating, calcination and post-heating in sequence, in which 400 °C was chosen as the calcination temperature. The pre-heating and post-heating temperatures were set at 250 and 350 °C, respectively. The droplets in the reactor underwent solvent evaporation, solute precipitation, precursor decomposition and conversion into Ag particles. The resulting powders were then collected using a cylindrical electrostatic collector with an applied high-voltage potential of −16 kV.

The prepared nanoparticles (NPs) were examined using transmission electron microscopy (TEM) and UV-vis spectroscopy. The submicron-particles (SMPs) were observed using scanning electron microscopy (SEM).

Ag particles of two different sizes and copper formate were mixed well to form composite pastes. There were two ingredients in the nanocomposite pastes. The first was 25 wt% for Ag NPs, 25 wt% for Ag SMPs, 20 wt% for copper formate and 30 wt% for α-terpineol, and the second was 25 wt% for Ag NPs, 25 wt% for Ag SMPs, 20 wt% for copper formate, 20 wt% for α-terpineol and 10 wt% for ethyl cellulose. α-terpineol was the solvent and ethyl cellulose acted as the thickener. The above hybrid pastes were named 6C-LNCP and 6C-LNCP(EC), respectively.

### 2.2. Bonding Process

Nanocomposite composite pastes were applied to the polished faying surface of a 5 × 5 × 1 mm^3^ Cu block, which was set onto a 12 × 12 × 3 mm^3^ Cu block to provide rigid joints. The Cu-to-Cu rigid joint specimens were bonded using the composite pastes with a thermal compression bonder in a N_2_ atmosphere. In addition to Cu, Ni/Au, and Ag surface finishes were also adopted to clarify the substrate effect. Ni/Au was prepared by electroplating onto Cu blocks, while the Ag coating was achieved using electroless deposition following the reaction below [20].

C_6_H_12_O_6 (aq)_ + 2Ag(NH_3_)_2_OH _(aq)_ → C_5_H_11_O_5_COONH_4 (aq)_ + 2Ag _(s)_ + 3NH_3 (aq)_ + H_2_O _(l)_

The bonding process comprised two ramp-soak steps. In the first step, the temperature was allowed to remain at 70 °C for 10 min to evacuate the organic components from the paste. After that, the samples were heated to 160 °C for 20 min under 1.6 MPa bonding pressure. FTIR was used to observe the residual organics in the LNCP sintered structure. To identify the structural phases, synchrotron radiation X ray diffraction (SR-XRD) observations were made on the sintered LNCP.

**Figure 1:**
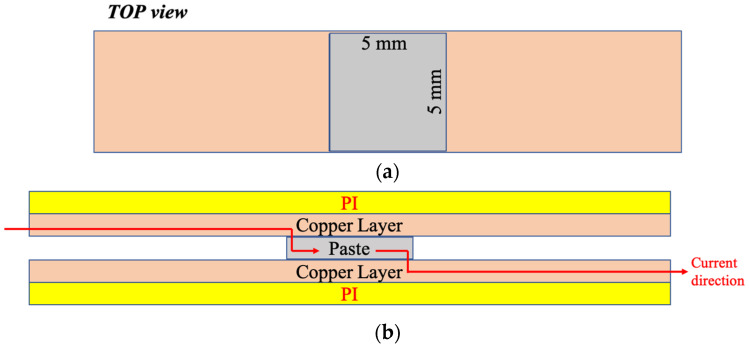
(**a**) Top and (**b**) side views of the two Cu-cladded PI substrates and the joint in-between, as well as the current stressing direction.

Cu foil-cladded PI substrates (PI: polyimide) were also used in this study (Figure 1). As shown, LNCP pastes were screen-printed onto copper pads with an area of 5 mmX5 mm. Some of the Cu pads were coated with a Ni/Au or Ag layer. Similar to the rigid samples (Cu blocks), the bonding process followed a two-ramp-soaking step with thermal compression bonding up to 160 °C (Table 1). The bonding pressure was increased to 10 MPa. For comparison, commercial bonding materials for flexible substrates, anisotropic conductive films (ACF) and silicone were also used to bond to the Cu foil-cladded PI substrate. As also noted in Table 1, the bonding conditions for ACF were 185 °C under 2 MPa for 20 s. The bonding conditions for silicone were room temperature under 2 MPa for 12 h.

### 2.3. Mechanical Testing for Rigid and Flexible Joints

The shear strength was measured by breaking both the rigid and flexible joints with a QC-506M1 bond tester (Cometech Testing Machines Co., Ltd., Taichung, Taiwan) shear tool with a shear rate of 0.2 mm/min. The reliability of the sintered joints on PI substrates was evaluated using the cyclic bending fatigue, thermal fatigue, and electrical current stress tests.

**Figure 2:**
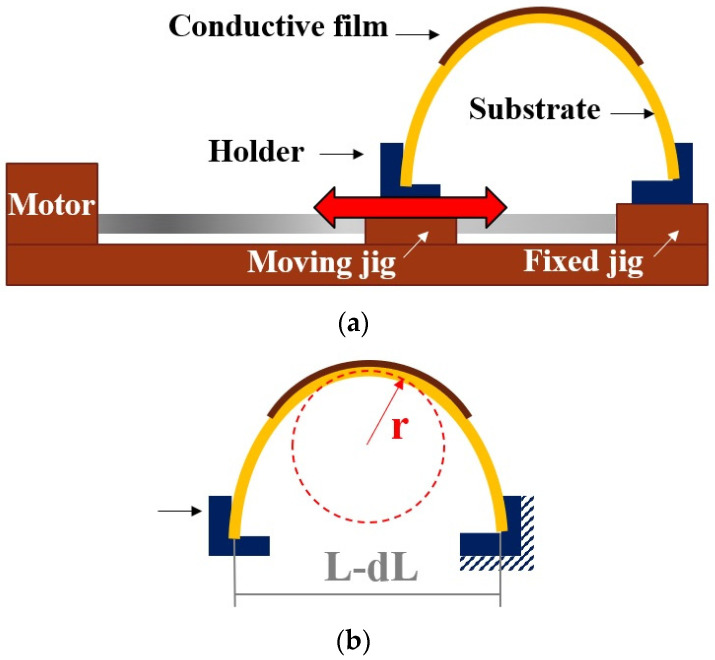
Bending experiment schematics: (**a**) bending apparatus (**b**) bending radius.

As illustrated in Figure 2a,b, the cyclic bending test was carried out at a frequency of 2 Hz for 1000 cycles. The outer bending radius of the PI film (R) was 4 mm, obtained according to Equations [21] (Figure 2b).

(1) $$R=\frac{L}{2\pi }\sqrt{\frac{dL}{L}-\frac{\pi^{2}h^{2}}{12L^{2}}}$$

where *L*, *dL/L* and *h* denote the initial length, the applied strain and the substrate thickness, respectively. As for thermal cycling, the temperature range was set between 40~85 °C. The soaking time was 15 min and ramping rate was 15 °C/min. Under a constant voltage of 5.4 V, flexible joints were charged with electric current from 0 A to 3 A, with an increase of 0.5 A for each test, as indicated in Figure 1. Electrical resistivity corresponding to the bending or thermal cyclic numbers was recorded, as well as current stressing time.

## 3. Results and Discussion

### 3.1. Ag Particle Characterization

**Figure 3:**
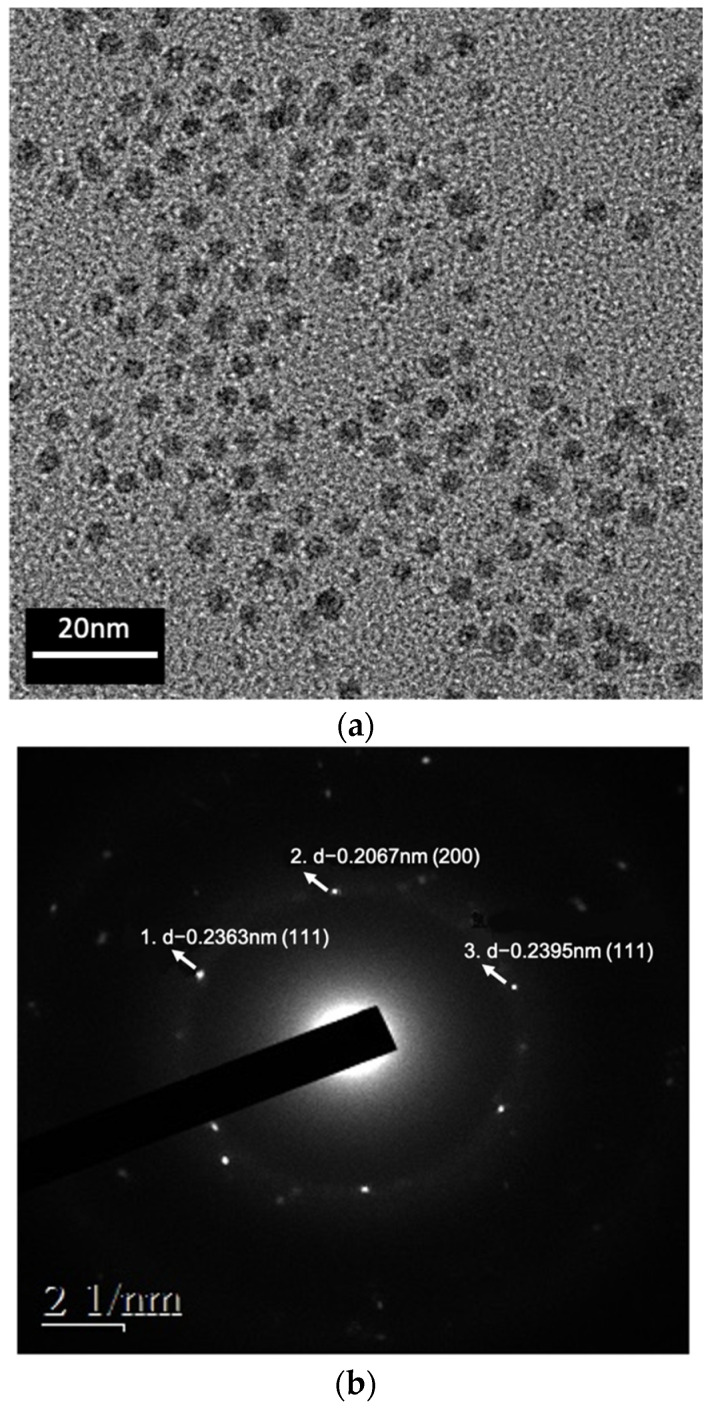

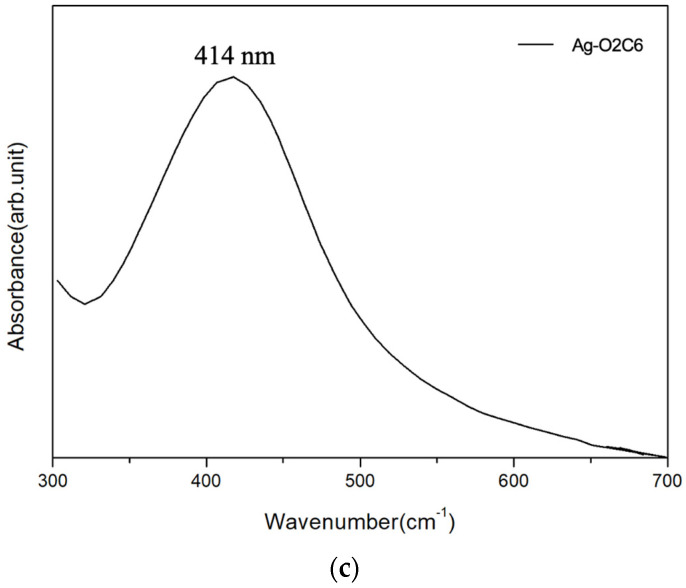
(**a**) TEM image and (**b**) diffraction patterns of Ag NPs, as well as (**c**) UV-vis spectrum.

**Figure 4:**
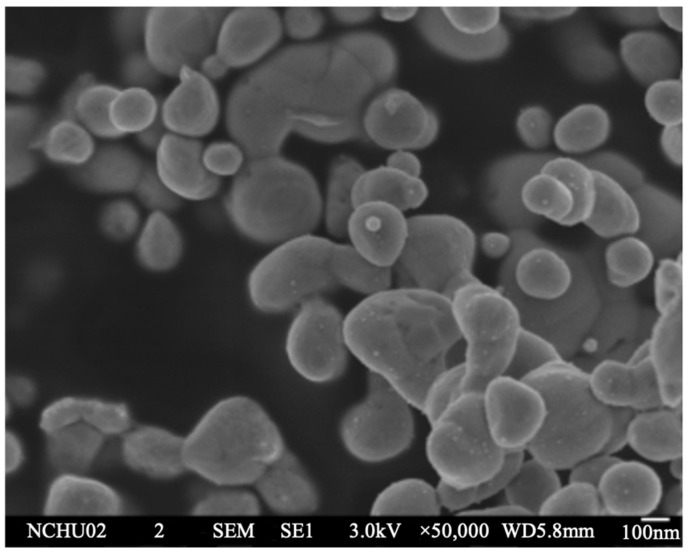
SEM image of spray pyrolyzed submicron Ag particles.

TEM image and diffraction patterns of the Ag NPs are shown in Figure 3a,b. The average diameter of Ag-O2C6 nanoparticles was 6.0 ± 1.0 nm. The PVP additive provided sufficient nanoparticle dispersion, allowing us to determine that the nanoparticles possessed uniform size. The ring diffraction patterns (see Figure 3b,d) verify the pure FCC Ag structure. The NPs could also be identified by the absorption spectra shown in Figure 3c. As illustrated, Ag-O2C6 NPs exhibited plasmon absorption bands with maxima at 414 nm, identical to the reported values. Figure 4 shows the morphology of the submicron particles (SMP). The average size was about 250 nm.

### 3.2. LNCP Characteristics and Sintered Structure

**Figure 5:**
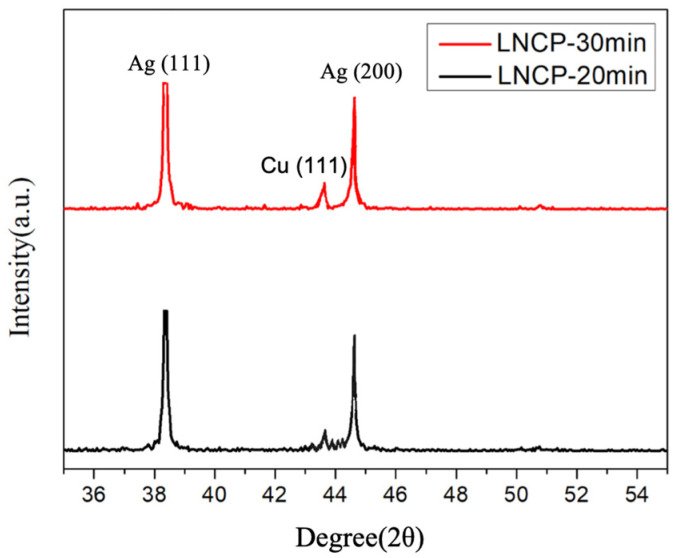
SRXRD patterns of LNCP subjected to isothermal heating at 160 °C for 20 or 30 min.

Figure 5 illustrates the SR-XRD spectra of LNCP sintered structures subjected to heating at 160 °C for 20 or 30 min, respectively. The major components are Ag and a small amount of Cu, detected without oxide formation.

**Figure 6:**
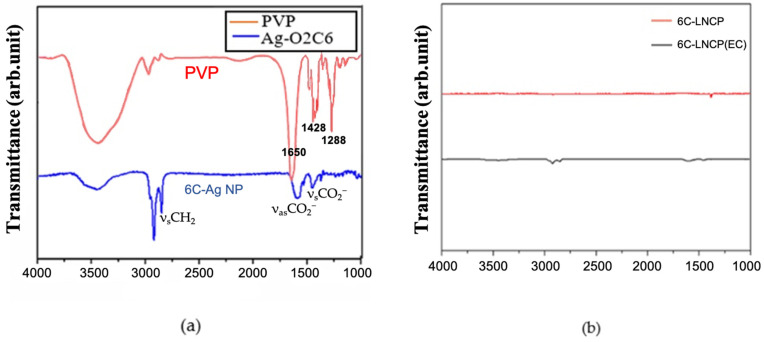
FT-IR spectra: (**a**) PVP (red) and Ag-6CO12 (blue) nanoparticles and (**b**) 6C-LNCP (red) and 6C-LNCP (black) after sintering.

The organic residues in the LNCP sintered structure were examined by FTIR. Figure 6a shows the FTIR spectra of PVP and the six carbon-chain-carboxylate-protected Ag NPs before sintering. The large band observed between 3750 cm^−1^ and 3000 cm^−1^ was linked to O-H stretching. The major PVP peaks were 1650 cm^−1^, 1428 cm^−1^, and 1288 cm^−1^. As also indicated in Figure 6a, ν_as_CH_2_, ν_s_CH_2_, ν_as_CO_2_^−^, and ν_s_CO_2_^−^ stretching peaks were detected [22]. After being subjected to thermal compression at 160 °C for 30 min, there was almost nothing left of the sintered LNCP (red curve), as shown in Figure 6b. Meanwhile, very tiny peaks were observed on the black curve (sintered 6 C-LNCP with ethyl cellulose), which were attributed to C-H and CO_2_^−^ stretching.

### 3.3. Joint Strength

**Figure 7:**
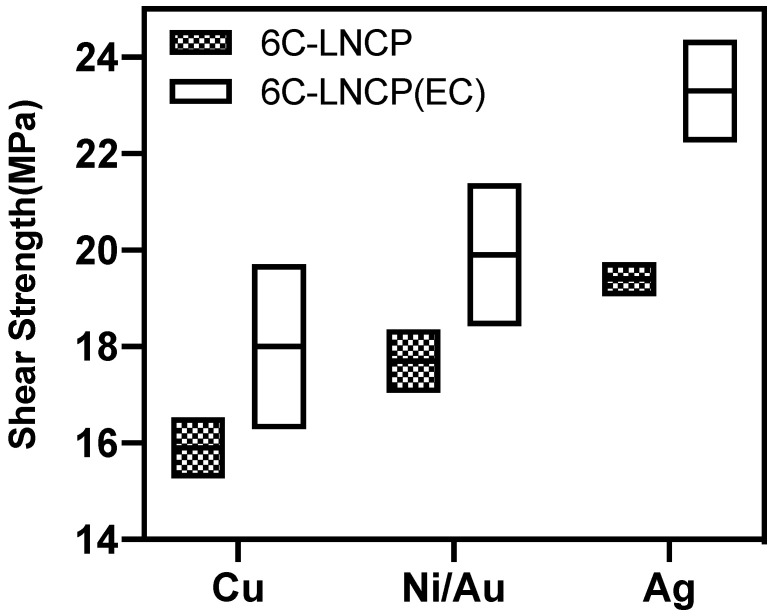
Shear strength of LNCP-6C joints with Cu, Ni/Au and Ag substrates, respectively (EC: ethyl cellulose).

**Figure 8:**
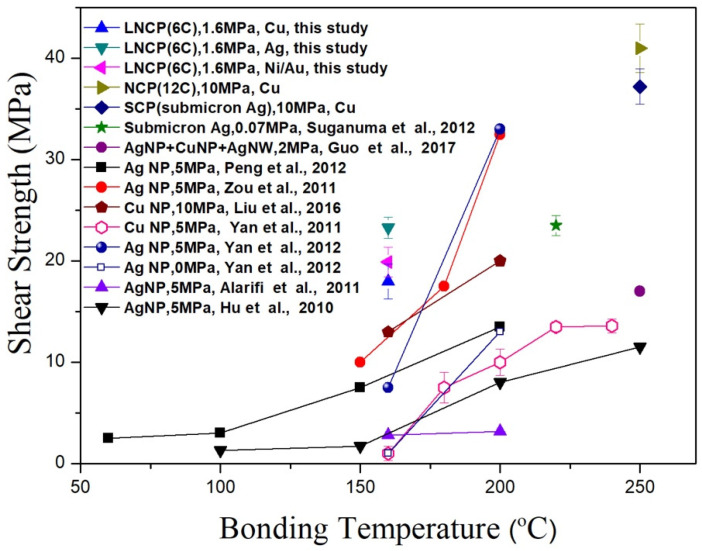
Comparison of strengths of joints bonded using NP pastes.

Figure 7 illustrates the joint shear strength with different substrates. AFfor 6C-LNCP pastes, the joint strengths were 15.8 MPa for the Cu substrate, 17.7 MPa for Ni/Au, and 19.2 MPa for Ag. It can be clearly seen that the addition of ethyl cellulose enhanced sintering and thus bonding strength. The joint strength for 6C-LNCP(EC) pastes were 18 MPa, 19.9 MPa and an eye-catching 23.3 MPa for Cu, Ni/Au and Ag, respectively. Figure 8 compares our work with the relevant literature [8,9,23,24,25,26,27,28,29], revealing that joints bonded using 6C-LNCP (EC) possessed superior shear strength to those using Ag or Cu NP pastes if the bonding temperature was set as 150 °C or 160 °C.

**Figure 9:**
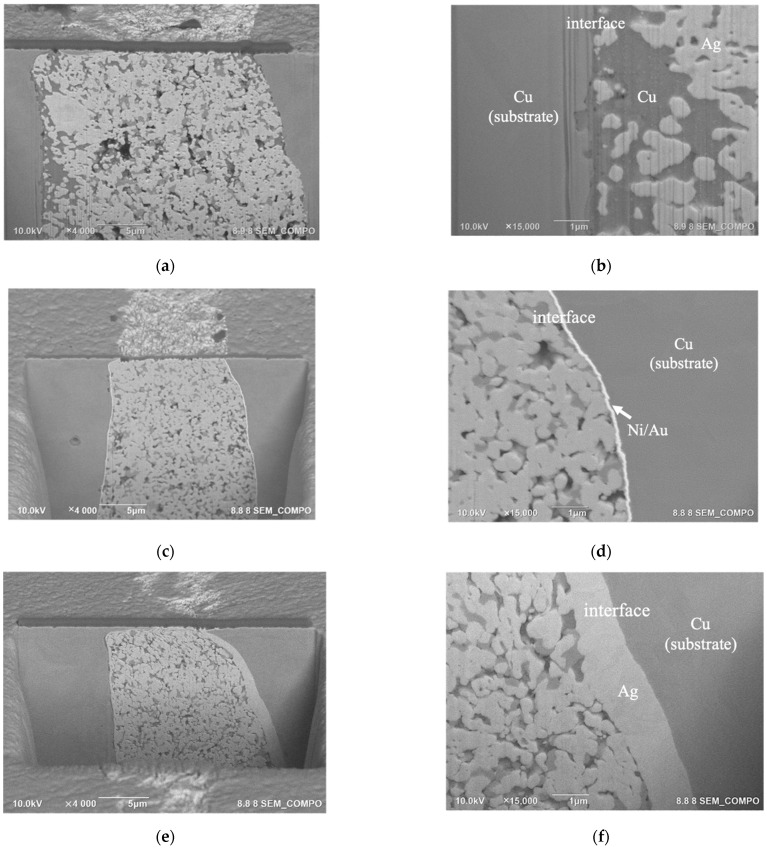
Cross-sectional LNCP(EC) joint microstructure with different substrates: (**a**,**b**) Cu, (**c**,**d**) Ni/Au and (**e**,**f**) Ag.

**Figure 10:**
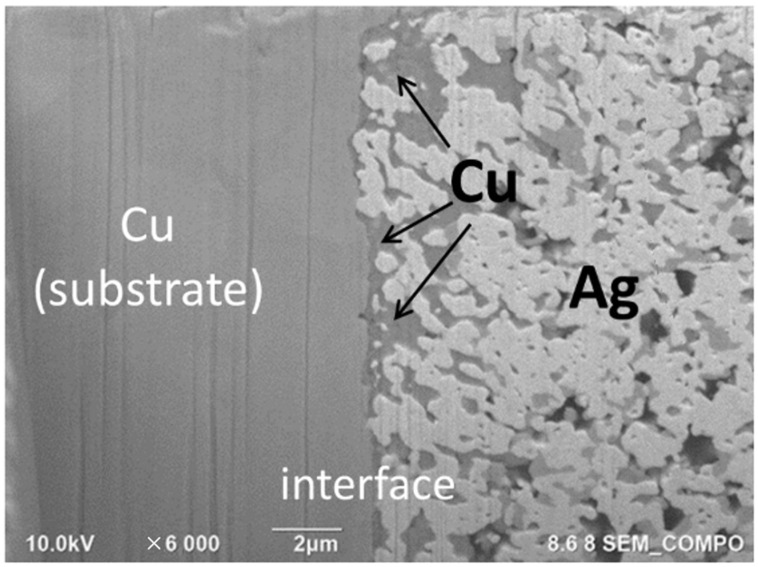
Cross-sectional LNCP(EC)/Cu joint microstructure from another location of the LNCP(EC)/Cu substrate joint (segregated Cu regions are indicated).

Figure 9 shows a cross-section of the LNCP(EC) joints, machined using a focused ion beam. All samples exhibited 4 to 7% porosity. The Cu joints showed a unique structural characteristic, that is, Cu tended to segregate in the vicinity of the joint interface (Figure 9c); there was no such Cu segregation for the Ni/Au and Ag substrate-joints. The images given in Figure 10 again verify that Cu segregated at the interface of LNCP(EC)/Cu interface. Considering the thermodynamic properties, the Cu-Ag binary system exhibited a highly positive mixing enthalpy $\left(\Delta H=+104\frac{\mathrm{meV}}{\mathrm{atom}}\;\mathrm{at}\;50\mathrm{at}\%\mathrm{Cu}\right)$ [30], a large lattice mismatch of 12% [31], and a limited electronegativity difference (0.03) [32]. This gave rise to the phase separation feature between Cu and Ag, i.e., Cu and Ag separated instead of dissolving into each other or forming intermetallic compounds. This drove the Cu atoms inside the paste to diffuse toward the Cu substrate. In addition, we also observed that the LNCP sintered structure linked well with the Ag substrate, which is why the Ag substrate joints possessed superior bonding strength.

### 3.4. Reliability Performance

**Figure 11:**
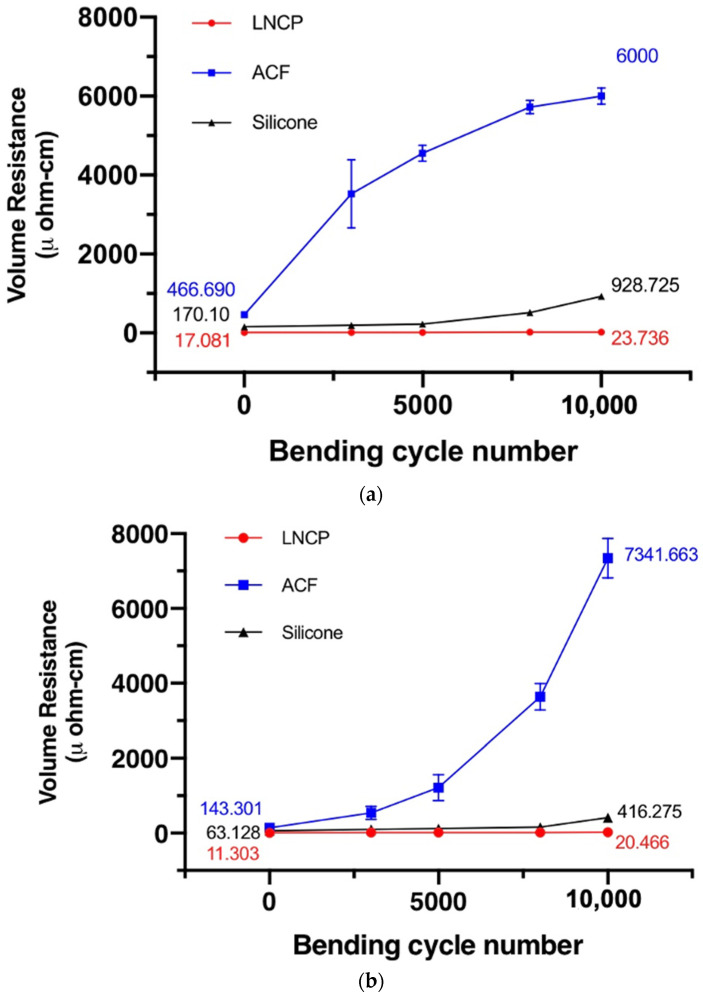
The relationship between the number of bends for test pieces and their respective electrical resistivity: (**a**) PI/Cu, and (**b**) PI/Cu/Ag.

**Figure 12:**
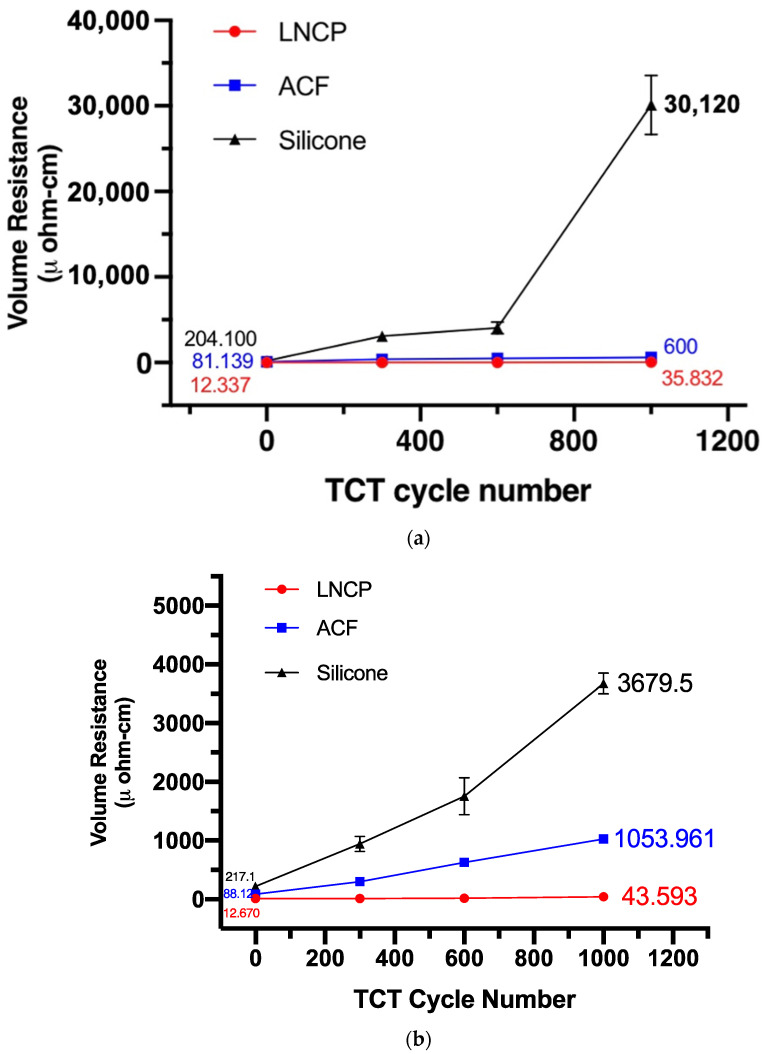
The relationship between the number of thermal cycles and respective electrical resistivity of the joints: (**a**) PI/Cu, and (**b**) PI/Cu/Ag (TCT: thermal cycling test).

**Figure 13:**
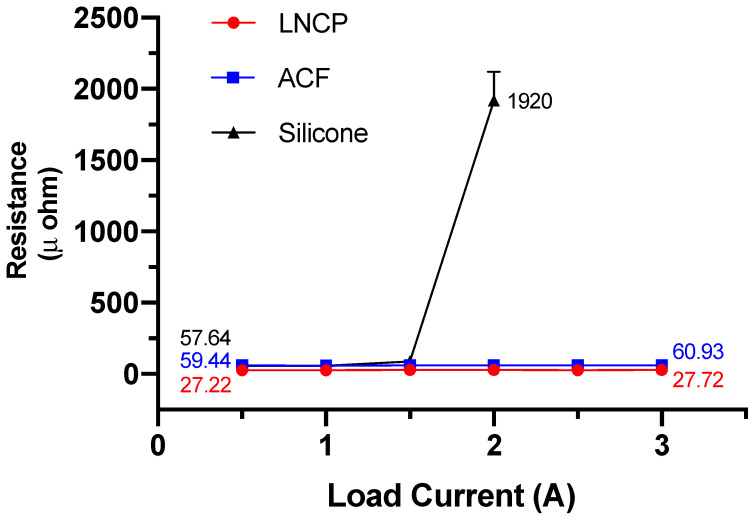
The relationship between applied current and the electrical resistivity of the joints with Cu substrate.

The reliability test results are given in Figure 11, Figure 12 and Figure 13. The electrical resistivity of the as-prepared LNCP joints was around 11~17 μΩ-cm, i.e., much lower than that of silicone joints (63~219 μΩ-cm) and ACF joints (81~466 μΩ-cm). The excellent electrical conductance again revealed that the LNCP did not suffer from the oxidation problem. Subjected to bending fatigue (Figure 11) or TCT cycles between −45~85 °C (Figure 12), the electrical resistivity of LNCP joints did not increase too much. The same tendency could be also observed in the current stressing test, in which the current was increased from 0.5 A to 3 A (Figure 13). As also revealed, the ACF electrical resistivity rose significantly when subjected to cyclic bending deformation, while the electrical conductance of silicone joints decayed drastically in the TCT and current stressing test. The above results led us to believe that ACF was vulnerable when repeated mechanical strain was applied, while the silicone was easy to damage through temperature variations.

It is interesting that the LNCP joints exhibited excellent performance, regardless of whether they suffered mechanical deformation, thermal deformation (due to CTE mismatch), or Joule heating resulting from current stressing. Even though the Ag-coated substrate has been recommended for Ag-based sintering [33] and also supported better shear strength, as mentioned in Figure 7, it did not behave better than a bare Cu substrate in this study when mechanical bending or TCT tests were involved. This could be ascribed to the effect of the addition of copper formate. Cu segregation may strengthen the interface and thus enhance the fatigue life of flexible interconnections.

## 4. Conclusions

Newly developed Cu-Ag composite pastes consisting of carboxylate-capped Ag nanoparticles, spray-pyrolyzed Ag submicron particles and copper formate were adopted in this study for bonding at low-temperatures and low bonding load. The joints between Cu, Ni/Au and Ag finished substrates could be well formed at temperatures as low as 160 °C under a load pressure of 1.6 MPa. The joint shear strength with Ag substrates reached 23 MPa, while those of Ni/Au and Cu were 19.9 MPa and 18 MPa, respectively. This nanocomposite paste could also be applied to form interconnections between metal-coated polymeric substrates. With respect to the joints between two Cu or Ag/Cu cladded PI substrates, electrical resistivity down to 11~17 μΩ-cm was obtained after thermal compression at 160 °C under a load pressure of 10 MPa for 20 min. Such flexible joints exhibited excellent tolerance when subjected to bending fatigue, thermal cycles or current stressing. The reliability performance was superior to that observed with joints created using commercial adhesives, e.g., ACF and silicone.

## Figures and Tables

**Table 1:** Processing conditions for bonding.

Bonding Materials	Bonding Condition					
	Temporary Bonding			Main Bonding		
	Temperature	Pressure	Time	Temperature	Pressure	Time
6C-LNCP 6C-LNCP (EC)	70 °C	10 MPa	600 s	160 °C	1.6, 10 MPa	1200 s
ACF	80 ± 10 °C	1 MPa	5 s	185 ± 10 °C	2 MPa	20 s
Silicone paste				Moisture	2 MPa	12 h

## Data Availability

The raw/processed data required to reproduce these findings cannot be shared at this time due to technical or time limitations.
